# Supplementary material for: Assessment of Stress and Immune Gene Expression in Australasian Snapper (Chrysophrys auratus) Exposed to Chronic Temperature Change
Source: Genes (Basel). 2025 Mar 28;16(4):385. doi: 10.3390/genes16040385 (PMC12027476; doi:10.3390/genes16040385)
Supplement: Supplementary file 1 [file genes-16-00385-s001.zip › genes-3507978-supplementary.pdf]

Supplementary Table S1. Gene targets analysed by NanoString Technologies, Inc.

Gene Name	GenBank Accession Number	Target Region	Target Sequence	HUGO Gene
Reference genes				
Elongation factor 1-α (*ef1a*)	Ch_aur001.1	1152–1251	CAAGAAGCTTGAGGATGCTCCCAAGTTCGTCAAGTCTGGTGATGCCGCCATTGT CAAACTGCACCCACAGAAGCCCATGGTTGTGGAGCCCTTCTCCAGC	LOC115578802
60S ribosomal protein L8 (*rpl8*)	Ch_aur022.1	634–733	GGTGGTGGTAACCATCAGCATATTGGCAAACCCTCAACAATCAGAAGGGACGC ACCTGCTGGTCGCAAGGTCGGTCTCATTGCTGCCCGTCGTACAGGCA	rpl8
40S ribosomal protein S18 (*rsp18*)	Ch_aur036.1	184–283	GAGGTTGAGCGTGTGGTGACCATCATGCAGAATCCTCGCCAGTACAAAATCCC AGACTGGTTCCTCAACAGGCAGAAGGACGTCAAGGACGGCAAATACA	LOC115577508
Target genes				
Actin cytoplasmic 1 (*actb*)	Ch_aur069.1	736–835	CAGGTCATCACCATCGGCAATGAGAGGTTCCGTTGCCCAGAGGCCCTCTTCCAG CCTTCCTTCCTCGGTATGGAGTCCTGCGGAATCCACGAGACCACCT	actb
Catalase isoform X2 (*cat*)	Ch_aur025.1	641–740	GCTACGGCTCTCACACCTTCAAACTGGTCAATGCCAATGGTGAGCGTTTCTACT GCAAGTTCCACTACAAGACTGATCAAGGAATAAAGAATCTGACAGT	cat
Cell surface glycoprotein MUC18-like isoform X (*muc18-like*)	Ch_aur003.1	412–511	ACTTACTTTGTTCCTGGAGGAACCAGGATGACTGAGACCAACCGTATTAACATC ACTGTATACTACCCCTCCACCGCTGTAAGTGTTTGGGTGGAGTCAC	LOC115570482
Complement C3-like A (*c3-like*)	Ch_aur061.1	2871–2970	TCTGATTCTCAATGCACAGCAACCTGACGGCATGTTTAAAGAAGTTGGAACGGT CTCCCACGGGGAGATGATTGGCGATGTGCGCGGCGCAGATTCAGAT	LOC115582848
Complement component C8 α chain (*c8a*)	Ch_aur047.1	568–667	TGGAGGAAATTCAGCTATGACTCATTCTGTGAGAACCTGCACTACAATGAAGA TGAGAAGAACTACAGGAAACCTTACAACTACCACACCTACCGTTTTG	c8a
Cryptochrome-1-like (*cry1-like*)	Ch_aur034.1	1655–1754	ACCAACAAACCAGCATCGGAACACACCAGCAAGGTTATCCAGGTACCAGTGC CGGTGTGATGTGTTACACTCAAGGCACACCACAGCAGATTCCTGGTTC	LOC115595869
Glutathione reductase mitochondrial isoform X2 (*gsr*)	Ch_aur019.1	236–335	TCAATGTTGGCTGTGTCCCTAAGAAGGTTATGTGGAATGCTGCAAGTCACGCCG AGTATCTCCATGATCACAATGATTATGGCTTCGACGTTGGAAATGT	gsr
Glutathione S-transferase A (*gsta*)	Ch_aur021.1	284–383	AACTGGCAATGATGTACCAGCGCATGTTTGAGGGTCTCTCACTCAACCAGAAA ATGGCGGATGTCATCTACTACAACTGGAAGGTCCCAGAGGGAGAGAG	LOC115579480
Glyceraldehyde-3-phosphate dehydrogenase (*gapdh*)	Ch_aur068.1	303–402	CTTGAAGGGTGGTGCCAAGAGAGTCATCATCTCTGCACCCAGCGCCGACGCTC CCATGTTTGTCATGGGTGTCAACCATGAGAAGTACGACCATTCCCTC	gapdh
Heat shock cognate 70 kDa protein (*hsp70*)	Ch_aur056.1	1605–1704	GGTGTCTGCTAAGAATGGCCTGGAGTCGTATGCTTTCAACATGAAGTCTACTGT GGAGGATGAAAAACTTGCTGGCAAAATCAGTGATGACGACAAGCAG	LOC115594641

heat shock protein HSP 90-β isoform X2 (*hsp90ab1*)	Ch_aur007.1	514–613	GGAGCTGACATCTCCATGATTGGTCAGTTTGGTGTGGGTTTCTACTCTGCCTACC TTGTTGCTGAGAAGGTGGTCGTCATCACCAAACACAACGATGATG	hsp90ab1
Hepcidin (*hamp*)	Ch_aur008.1	101–200	AGGAGGCAGGGAGCAATGACACTCCAGTTGCGGCACATCAAGAAATGTCAAT GGAATCGTGGATGATGCCGAGTCGCGTCAGGGAGAAGCGTCAGAGCCA	hamp
Insulin-like growth factor I isoform X1 (*igf1*)	Ch_aur033.1	188–287	GAGAGAGAGGCTTTTATTTCAGTAAACCTGGCTATGGCCCCAATGCACGGCGGT CACGTGGCATTGTGGACGAGTGCTGCTTCCAAAGCTGTGAGCTGCG	igf1
Insulin-like growth factor II (*igf2*)	Ch_aur050.1	161–260	CGCTGTGTGGGGGAGAGCTGGTGGATGCGCTGCAGTTTGTCTGCGAAGACAGA GGCTTCTATTTCAGTAGGCCAACCAGCAGGGGAAACAACCGGCGCCC	igf2
Mitochondrial uncoupling protein 2-like (*ucp2-like*)	Ch_aur038.1	539–638	TCACTAGAAATGCGCTTGTCAACTGCACAGAACTGGTTACATACGACCTGATCA AGGAGGCCATCCTCAAACACAACCTGTTGTCAGACAACCTGCCCTG	LOC115579854
Nuclear factor erythroid 2-related factor 2 (*nrf2*)	Ch_aur065.1	1360–1459	CAGAGGGCTAAGGCCCTCAAAATCCCTTTCACTGTAGACATGATTATCAATCTG CCTGTCGACGATTTCAATGAGCTGATGTCAAAGCACCGACTGAATG	nfe2l2
Peroxiredoxin-1 (*prdx1*)	Ch_aur042.1	162–261	CGAGATCATAGCTTTCAGTGACGCTGCTGACGATTTCAGGAAGATCGGCTGTGA GGTCATCGCCGCCTCTGTTGACTCACACTTCTCCCATTTCGCATGG	LOC115573364
Peroxiredoxin-1-like (*prdx-like*)	Ch_aur013.1	374–473	CATACAGGGGGCTGTTTGTGATTGACGACAAGGGCATCTTGAGGCAGATCACC ATCAATGACTTGCCTGTGGGTCGCTCTGTGGATGAGACTCTGCGCCT	LOC115587998
Peroxiredoxin-5 mitochondrial (*prdx5*)	Ch_aur057.1	152–251	TGTCTATGGATCAGCTCTTCAAGGGGAAGAAGGGAGTCCTCTTTGCTGTACCTG GAGCCTTCACACCTGGATGTTCCAAGACTCACCTCCCAGGTTTTGT	prdx5
Prostaglandin G/H synthase 2 (*ptgs2*)	Ch_aur071.1	1187–1286	TCGTCTTCAACACGTCTGTAGTGACTGAGCACGGCATCAGCAACCTTGTGGAGT CGTTTTCCAAGCAGATCGCTGGACGGGTTGCCGGTGGCCGAAATGT	ptgs2
Serotransferrin-like (*tf-like*)	Ch_aur005.1	582–681	CGAGCCTTATTATGACTACGGTGGAGCCTTCCAATGTCTGGCAGACGACGCTGG TGATGTGGCCTTTGTGAAGCATCTCACTGTACCTGAGTCTGAAAAG	LOC115572354
Superoxide dismutase [Cu-Zn] (*sod1*)	Ch_aur059.1	100–199	GGAGAAATCTCGGGACTTACTCCTGGTGAGCATGGTTTCCATGTCCATGCATTT GGAGACAATACAAATGGGTGCATCAGTGCAGGCCCTCACTTCAATC	sod1
Suppressor of cytokine signalling 3 (*socs3*)	Ch_aur039.1	291–390	GCGCATCCAGTGTGACTCAAGCTCTTTTTTCCTGCAGACGGACCCTAAAAACGT TCAGTCTGTTCCTCACTTTGACTGCGTCCTCAAGCTGGTGCATTAC	socs3
Thioredoxin-dependent peroxide reductase mitochondrial (*prdx3*)	Ch_aur006.1	290–389	CCTTTGTGTGTCCAACAGAGATCATCTCATTCAGCGACAAGGCCAGTGAGTTCC ACGACGTTAACTGTGAGGTGGTGGGTGTGTCGGTGGACTCTCACTT	prdx3
Transforming growth factor β-1 proprotein-like isoform X1 (*tgfb1-like*)	Ch_aur024.1	300–399	CAGTGCCATCAATTTTGAGGTCTCCGGGATCTCGAATAGTAGGGGAGACACAC AAGGGTTTCAACAGGTGTCGCAGCAATACCCGTACATCCTGACCATG	LOC115575711
Genes that did not pass calibration				

Alkaline phosphatase tissue-nonspecific isozyme isoform X1 (*alpl*)	Ch_aur035.1	661–760	GGCTGCAAGGATATCGCCAGACAACTCTTTGAAAATATTCCCAACATTGATGTG ATTATGGGTGGAGGAAGGAAGTATATGTTCCCTAAAAACAAGTCGG	alpl
Complement component C6 (*c6*)	Ch_aur018.1	2413–2512	CTCTGTATCCTGAACGTAGACCTCGGCGTCACCGTGCCGATGTCCCTCTGCTCCT TCCACGTCGGGCTTTGCCACAATGATCCGCTCTTCTATGTCAGCG	c6
Glucocorticoid receptor-like (*gcr-like*)	Ch_aur049.1	669–768	GGACGTTGGCTCAGAGAGGGACATGAAGTCTGCTGTGGTTGAAAGCATTAACG GCAGTGGGGCAGTCTTTGTTGCTCTTAATGGCAGTAATATGACAAGT	LOC115568693
Glutathione peroxidase 2 (*gpx2*)	Ch_aur064.1	103–202	TGTGGCCTCGCTCTGAGGCACCACCACCCGGGACTACAGCGAGCTCAACCAGC TGCAGAGCAAGTACCCGCATCGGCTGGTGGTCCTGGGTTTTCCCTGT	gpx2
Hypoxanthine-guanine phosphoribosyltransferase (*hprt1*)	Ch_aur031.1	253–352	CTGAACAGGAACAGTGACCGCTCCATCCCAATGACAGTGGACTTCATCCGCCT CAAGAGCTACTGTAACGACCAGTCGACAGGTGAAATCAAAGTGATTG	hprt1
Interferon-induced GTP-binding protein Mx-like B (*mx-like*)	Ch_aur051.1	982–1081	CCATCTGATGCAGCTGAGAGAGTCGTCTTCCTCATTGATAAAGTGACAGCTTTC ACTCAGGATGCCATCAGTCTGACCACAGGAGAAGAACTCAATTGTG	LOC115583120
Interleukin-1 β-like (*il1b-like*)	Ch_aur029.1	412–511	CCTACACCCAGTGCTGAGGCCGTAACTGTGACTCTGTGCATCAAGGACACAAA TCTTTACCTGTCTTGTCACAAGGAAGGTGACGAGCCAACCTTGCATC	LOC115581181
Interleukin-6 isoform X1 (*il6*)	Ch_aur045.1	127–226	GTGATGCTGGCCGCTCTGCTTCAGTGTGCTCCCGGGGCTCCGATTGATGGCGCG CTCACTGACAATCCAGCAGGTGACACCTCAGGTGAAGAGTGGGAGA	LOC115579128
Interleukin-10-like (*il10-like*)	Ch_aur062.1	528–627	AGGTCTATACAAGGCCATGGGAGAGCTGGATCTGCTGTTCAACTACATTGAGA CATATCTGGCTTCCAAACGGCACGGAACACATGTGGCCTCCGCTTGA	LOC115582730
Interleukin-12 subunit β-like (*il12b-like*)	Ch_aur032.1	388–487	GCACCTAACTATTCAGGCTCCTTCAAATGCACCTGGGCTAAAGCAGAGCACAG ATCCAACGCCGCCGTGCTCCTGGTGAAGGCCGAACGTCATTTGGAGA	LOC115593944
Interleukin-17D (*il17d*)	Ch_aur010.1	415–514	CGCAGCACTCCGGTCTACGCTCCGTCTGTCATCCTGAGGAGAACCGGCTCCTGC CTCGGCGGCCGACACTCATACACAGAGATCTACGTCTCCATCGCGG	il17d
Interleukin-34 isoform X1 A (*il34*)	Ch_aur009.1	142–241	CGGTACATGAGGCACAACTTCCCCATCAAGTACACCATCAGGGTTCATCACAA CGAAGTCTTTAAACTGTCAAACATCAGCAGACTGAGGTTACAGGTGG	il34
Macrophage colony-stimulating factor 1 receptor (*csf1r*)	Ch_aur060.1	2343–2442	CAAAAATTGTATTCACAGAGACATCGCTGCAAGGAATGTCCTGTTGACTGATCA CAGAGTGGCCAAGATTTGTGACTTTGGTCTGGCACGTGACATCATG	csf1r
Mucin-2-like isoform X1 (*muc2-like*)	Ch_aur020.1	1740–1839	CTGTTCCCTCAGTGTGGAAAATGAGAATTACGCCAAACACTGGTGTGCCTTGCT GCTAAGTCCAGACAGCTCCTTTGCACAGTGCCGTTCAGCGGTGGAT	LOC115586438
Nuclear factor NF-kappa-B p100 subunit isoform X1 (*nfkb2*)	Ch_aur030.1	717–816	GGAGGCGTTCGGAGACTTTTCACCAACCGACGTTCACAAACAGTACGCCATTGT GTTCAAAACGCCGCCCTATCACAGCGCAGAGATCGAGCGGCCCGTC	nfkb2
Stromal cell-derived factor 1 (*cxcl12*)	Ch_aur016.1	197–296	AGAACAACAGGGAAGTTTGCATCAACCCGGAGACCAAGTGGCTGCAGCAGTA CTTAAAGAACGCCATTAACAAGGTGAAGAAAAACCGAAGACGCAATAA	cxcl12

T-cell surface glycoprotein CD8 α chain-like isoform X3 (*cd8a-like*)	Ch_aur017.1	151–250	TGGTTTCGAGTGCTGGACAAATCTGGCATGGAATTCATTGGGTCTTTCAGCAAT ACTGGCGTGAAAAAACCAAATACAAAGCCTCCAACTTCCATCTTCA	LOC115583354
Toll-like receptor 2 isoform X1 (*tlr2*)	Ch_aur048.1	602–701	CGAGGTATGAGTCCGGTACTCTGGCATACGTTTGGCCGTTGGGTCGTGTCACTTT GAGCCTCCACAGTCCATTTTTAACAAATGAGGCCTTAGCCTCAGC	LOC115590525
Toll-like receptor 3 (*tlr3*)	Ch_aur014.1	799–898	AGCCAAGCTGATGGCAGCTTTCAGCCGTACAGCGCGGTGCTGCAGACCACTGA ATCACTCAAAGTACTTCAGCTGCAATTCATGAAGGTGTTGATAGAAA	LOC115590587
Toll-like receptor 5 (*tlr5*)	Ch_aur053.1	1203–1302	CTTCCCTGCGTCTCTACCCAGATTAGATTATCTCCTGTTGAACGACAACAAGTTG ACCTCCTCGTCAGTATACAGTCTCACACGGTTTGCCGATAATGCC	LOC115574263

Supplementary Table S2. Mean gene counts (n=10) with standard errors (SE) for snapper exposed to either warm (22°C), cold (14°C) or ambient temperatures for 3 months.

Gene	Tissue	Statistic	Warm	Cold	Ambient
Actin cytoplasmic 1 (*actb*)	Fin clip	Mean	9833.3*	7353.7	6952.2
		SE	281.7	196.5	379.8
	Liver	Mean	9535.3*	5307.6*	3643.3
		SE	765.1	357.5	148.2
	Head kidney	Mean	20715.0	13404.4*	17038.3
		SE	560.0	612.2	874.6
Catalase isoform X2 (*cat*)	Fin clip	Mean	59.2	48.4	60.0
		SE	2.3	5.2	3.3
	Liver	Mean	2666.1*	2192.4*	1101.7
		SE	231.9	160.8	41.7
	Head kidney	Mean	691.9*	282.4	232.7
		SE	210.3	12.4	24.8
Cell surface glycoprotein MUC18-like isoform X (*muc18-like*)	Fin clip	Mean	209.1*	90.3	79.9
		SE	13.4	7.4	4.3
	Liver	Mean	406.7*	207.3*	122.9
		SE	39.0	14.1	6.5
	Head kidney	Mean	461.8	145.9	156.7
		SE	238.5	10.7	20.2
Complement C3-like A (*c3-like*)	Fin clip	Mean	18.9	32.0*	10.2
		SE	4.7	10.2	1.7
	Liver	Mean	38563.3*	27545.5	16684.0
		SE	3096.5	3153.1	1598.6
	Head kidney	Mean	268.6*	2.5	2.3
		SE	262.2	0.3	0.4
Complement component C8 alpha chain (*c8a*)	Fin clip	Mean	3.3*	6.8*	14.3
		SE	1.1	1.9	2.2
	Liver	Mean	4222.5*	2983.5	1711.0
		SE	240.4	200.9	126.9
	Head kidney	Mean	1316.6	2.3	1.8
		SE	1314.3	0.5	0.1
Cryptochrome-1-like (*cry1-like*)	Fin clip	Mean	273.7*	367.8	435.4
		SE	12.0	22.7	19.3
	Liver	Mean	170.8*	186.8*	101.4
		SE	14.4	14.8	3.6
	Head kidney	Mean	392.3*	195.1	233.0
		SE	246.8	8.3	16.1
	Fin clip	Mean	162.4	150.6	214.9

Glutathione reductase mitochondrial isoform X2 (*gsr*)		SE	10.8	8.1	9.8
	Liver	Mean	70.8	125.5	87.9
		SE	9.2	12.5	6.1
	Head kidney	Mean	561.6	345.0	325.2
		SE	228.0	8.7	19.2
Glutathione S-transferase A (*gsta*)	Fin clip	Mean	386.6	376.4	334.3
		SE	32.0	41.7	29.0
	Liver	Mean	5666.6*	2111.0*	514.1
		SE	1022.8	527.7	108.5
	Head kidney	Mean	591.8	241.0	236.0
		SE	233.6	24.0	27.5
Glyceraldehyde-3-phosphate dehydrogenase (*gapdh*)	Fin clip	Mean	24.2*	21.9*	5.8
		SE	3.2	5.8	1.6
	Liver	Mean	24548.5	16799.5	10728.7
		SE	1583.6	1283.8	607.6
	Head kidney	Mean	702.8	191.3	156.7
		SE	323.6	46.3	23.4
Heat shock cognate 70 kDa protein (*hsp70*)	Fin clip	Mean	9141.3*	16908.4*	21976.4
		SE	409.0	640.3	586.9
	Liver	Mean	8192.6*	11034.6	11162.7
		SE	473.0	652.8	443.5
	Head kidney	Mean	11152.9	13641.4	14144.0
		SE	820.2	257.6	334.5
Heat shock protein HSP 90-beta isoform X2 (*hsp90ab1*)	Fin clip	Mean	7216.7*	9221.3	9248.6
		SE	340.8	266.3	270.0
	Liver	Mean	10465.4*	8945.5*	7409.2
		SE	426.3	497.5	272.6
	Head kidney	Mean	8504.4*	6906.7	7172.9
		SE	533.6	169.1	255.8
Hepcidin (*hamp*)	Fin clip	Mean	8.4	14.4	13.3
		SE	1.8	3.3	2.8
	Liver	Mean	10665.9	7236.2	5528.7
		SE	1834.2	667.5	410.2
	Head kidney	Mean	554.7	14.0	30.9
		SE	522.3	3.8	14.4
Insulin-like growth factor I isoform X1 (*igf1*)	Fin clip	Mean	113.3	58.0	32.9
		SE	10.5	5.2	3.2
	Liver	Mean	1898.6	1884.2	1088.2
		SE	142.8	171.9	81.2

	Head kidney	Mean	299.9	4.5	13.9
		SE	259.7	2.5	5.0
Insulin-like growth factor II (*igf2*)	Fin clip	Mean	94.2*	103.2*	54.7
		SE	6.0	8.0	3.0
	Liver	Mean	2058.3*	359.8	373.9
		SE	165.9	42.6	108.1
	Head kidney	Mean	293.6	30.2	22.4
		SE	259.1	1.6	1.7
Mitochondrial uncoupling protein 2-like (*ucp2-like*)	Fin clip	Mean	4.3	10.2*	4.8
		SE	0.5	1.6	1.0
	Liver	Mean	3734.3	1566.5	1664.1
		SE	316.5	195.9	121.8
	Head kidney	Mean	262.9	3.5	0.0
		SE	262.9	2.5	0.0
Nuclear factor erythroid 2-related factor 2 (*nrf2*)	Fin clip	Mean	131.3	83.5	111.6
		SE	4.9	7.0	8.7
	Liver	Mean	279.7*	274.4*	147.4
		SE	31.9	20.3	11.0
	Head kidney	Mean	548.6	289.6	226.5
		SE	227.6	14.9	11.5
Peroxiredoxin-1 (*prdx1*)	Fin clip	Mean	153.8	187.8	254.9
		SE	4.1	16.5	24.1
	Liver	Mean	2197.6*	1767.7*	631.6
		SE	201.4	357.0	76.4
	Head kidney	Mean	1401.2	297.4	407.3
		SE	1008.1	19.1	17.9
Peroxiredoxin-1-like (*prdx*-like)	Fin clip	Mean	339.2	386.8	442.3
		SE	9.7	17.6	31.2
	Liver	Mean	1007.8*	620.1*	400.2
		SE	54.5	40.1	17.0
	Head kidney	Mean	1514.9*	1236.2	978.5
		SE	152.6	96.3	92.0
Peroxiredoxin-5 mitochondrial (*prdx5*)	Fin clip	Mean	330.9*	494.5	564.7
		SE	14.0	35.5	34.9
	Liver	Mean	78.1	128.4	96.4
		SE	5.8	9.4	5.1
	Head kidney	Mean	371.5	163.1	160.2
		SE	249.5	10.1	13.4
	Fin clip	Mean	102.6	93.5	195.7

Prostaglandin G/H synthase 2 (*ptgs2*)		SE	7.7	11.0	13.1
	Liver	Mean	50.2*	10.3	3.8
		SE	6.8	3.3	2.1
	Head kidney	Mean	270.7	2.6	0.0
		SE	262.0	2.8	0.0
Serotransferrin-like (*tf-like*)	Fin clip	Mean	26.2	67.7*	18.3
		SE	6.9	21.6	2.9
	Liver	Mean	72348.9	38649.1	35750.9
		SE	4256.3	2512.4	4664.5
	Head kidney	Mean	1316.5	0.0	0.0
		SE	1314.3	0.0	0.0
Superoxide dismutase [Cu-Zn] (*sod1*)	Fin clip	Mean	357.3*	418.2	517.7
		SE	13.0	24.7	35.5
	Liver	Mean	1637.1*	1265.4*	715.7
		SE	138.3	62.4	46.6
	Head kidney	Mean	949.9	430.4	428.9
		SE	473.9	22.7	20.8
Suppressor of cytokine signalling 3 (*socs3*)	Fin clip	Mean	363.1*	96.4	108.4
		SE	103.8	9.3	9.3
	Liver	Mean	92.3*	39.1	22.7
		SE	20.4	6.1	5.4
	Head kidney	Mean	531.0*	60.4	62.2
		SE	234.3	10.6	9.4
Thioredoxin-dependent peroxide reductase mitochondrial (*prdx3*)	Fin clip	Mean	119.8*	165.6*	288.0
		SE	7.2	9.0	17.0
	Liver	Mean	366.8	409.0	337.6
		SE	31.9	28.0	18.7
	Head kidney	Mean	1754.2	225.7	231.2
		SE	1555.4	12.2	16.0
Transforming growth factor beta-1 proprotein-like isoform X1 (*tgfb11-like*)	Fin clip	Mean	405.4*	268.1	268.5
		SE	24.3	16.0	12.1
	Liver	Mean	48.6*	43.1*	21.8
		SE	7.3	5.5	1.1
	Head kidney	Mean	457.0	214.4	190.8
		SE	238.9	14.0	5.9

Mean gene counts (n=10) are shown with standard errors for warm, cold and ambient (control) treatments. Significant difference from control using the Fisher's least significant difference test ($p = 0.001 = 0.05/(2*25)$) is indicated by *.

Supplementary Table S3. Correlations between tissue gene expression counts in individual fish tissues in both chronic-temperature experiments (data published here) and acute-temperature experiments (Published in Bentley-Hewitt et al. [1]).

Gene	Chronic experiment		Acute experiment	
	Fin clip vs Head kidney (r value)	Fin clip vs Liver (r value)	Fin clip vs Head kidney (r value)	Fin clip vs Liver (r value)
actb	0.62	0.78	-0.08	0.40
cat	0.02	-0.06	-0.66	-0.29
muc18-like	0.66	0.85	0.00	0.26
c3-like	0.06	0.33	0.06	0.17
c8a	-0.12	-0.72	-0.03	-0.13
cry1-like	0.63	-0.54	0.82	0.91
gsta	0.07	0.29	-0.02	0.46
gsr	-0.11	0.00	0.24	-0.15
hsp70	0.76	0.61	0.43	0.52
hsp90	-0.49	-0.49	0.81	0.83
hamp	0.21	-0.16	0.34	-0.03
igf1	0.18	0.61	0.47	0.41
igf2	0.28	0.36	0.14	0.00
ucp2-like	0.10	-0.15	-0.05	-0.18
nrf2	0.05	0.07	0.23	-0.15
prdx1	0.27	-0.29	0.57	-0.23
prdx1-like	-0.18	-0.59	-0.06	0.11
prdx5	0.51	0.52	0.60	0.56
ptgs2	-0.01	-0.40	-0.08	0.03
tf-like	-0.26	-0.15	0.10	-0.06
sod	-0.09	-0.49	0.24	0.27
soc3	0.73	0.48	0.18	0.13
prdx3	0.22	-0.12	-0.20	-0.28
tgfb1-like	0.14	0.40	-0.06	0.01
gapdh	0.06	0.70	-0.04	-0.29

Shows r values for gene correlations between fin clip and head kidney or liver in both chronic (n=30) and acute (n=50) experiments. Significant values p=0.01 are when r values > 0.46 (chronic experiment, n=30) or >0.36 (acute experiment, n=50).

1. Bentley-Hewitt, K.L.; Flammensbeck, C.K.; Crowhurst, R.N.; Hedderley, D.I.; Wellenreuther, M. Development of a Novel Stress and Immune Gene Panel for the Australasian Snapper (Chrysophrys auratus). *Genes* **2024**, *15*, 1390.

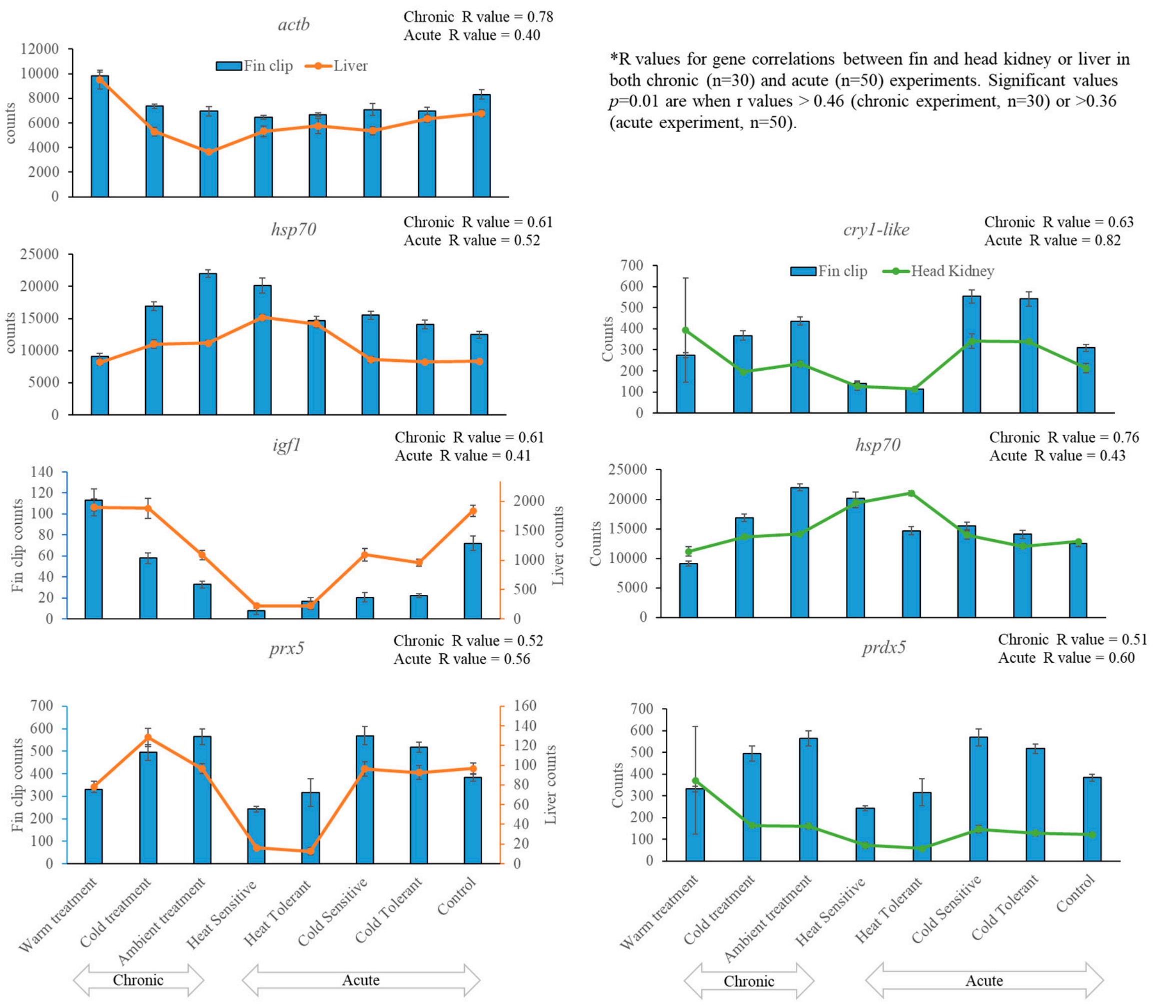

Supplementary Figure S1. Mean gene counts (n=10) from fin, liver (left side) and head kidney (right side) tissue are shown with standard errors for warm treatment, cold treatment and ambient treatment (chronic-temperature experiment) heat-sensitive, heat-tolerant, cold-sensitive, cold-tolerant and control (acute-temperature experiment), treated fish. In two instances, the scale of mean gene counts were different in liver compared with fin and the new scale is shown on the right axis of the graph. R values showing the correlation of fin expression with either liver or head kidney are shown in the top right of each graph in both chronic (n=30) and acute (n=50) experiments. Significant values p=0.01 are when r values > 0.46 (chronic experiment, n=30) or >0.36 (acute experiment, n=50).
